# Endoscopic Neck Surgery for Thyroid Carcinoma

**DOI:** 10.1155/DTE.7.135

**Published:** 2001

**Authors:** Hiroya Kitano, Takashi Kinoshita, Hideyuki Kataoka, Masamitsu Hirano, Eiji Takeuchi, Kazutomo Kitajima, Masaki Fujimura

**Affiliations:** 1 Department of Otolaryngology Head and Neck Surgery Shiga University of Medical Science Seta Otsu Shiga 520-2192 Japan; 2 Department of Surgery Shiga University of Medical Science Seta Otsu Shiga 520-2192 Japan; 3 Department of Otolaryngology Head and Neck Surgery Faculty of Medicine Tottori University, 36-1 Nishimachi Yongao Japan

## Abstract

In the past 5 years, endoscopic neck surgery has been performed by various surgeons in Japan. However, many problems remain to be solved, including indications for this related in malignant thyroid tumors. For small thyroid cancers and legions suspected of malignancy, we found that we could obtain radicality in endoscopic neck surgery that was comparable to that attainable by conventional methods. Here, we describe our recent endoscopic surgical experience in five patients with preoperative diagnoses of definite or suspected thyroid carcinoma.


*Diagnostic and Therapeutic Endoscopy*, Vol. 7, pp. 135–140
Reprints available directly from the publisher
Photocopying permitted by license only

© 2001 OPA (Overseas Publishers Association) N.V.
Published by license under
the Harwood Academic Publishers imprint,
part of Gordon and Breach Publishing
a member of the Taylor & Francis Group.
All rights reserved.

### Endoscopic Neck Surgery for Thyroid Carcinoma

HIROYA KITANO[a]*, TAKASHI KINOSHITA[b], HIDEYUKI KATAOKA[a], MASAMITSU HIRANO[b], EIJI TAKEUCHI[a], KAZUTOMO KITAJIMA[a] and MASAKI FUJIMURA[b]

[a]*Department of Otolaryngology, Head and Neck Surgery;* [b]*Department of Surgery, Shiga University of Medical Science, Seta, Otsu, Shiga 520-2192, Japan*

*(Received 14 May 2001; In final form 14 May 2001)*

In the past 5 years, endoscopic neck surgery has been performed by various surgeons in Japan. However, many problems remain to be solved, including indications for this related in malignant thyroid tumors. For small thyroid cancers and legions suspected of malignancy, we found that we could obtain radicality in endoscopic neck surgery that was comparable to that attainable by conventional methods. Here, we describe our recent endoscopic surgical experience in five patients with preoperative diagnoses of definite or suspected thyroid carcinoma.

*Keywords*: Endoscopic surgery; Laparoscope; Thyroid cancer

### INTRODUCTION

Endoscopic neck surgery using a laparoscope with a video camera first was performed by Gagner *et al.* who successfully carried out an endoscopic subtotal parathyroidectomy in 1996 [1,2]. Endoscopic neck surgery had not been attempted previously because of restricted working space in the neck. In laparoscopic surgery, space is created by gas insufflation; in endoscopic neck surgery, the narrow working space would need to be enlarged by bluntly dissecting subplatysmal tissues, while carbon dioxide gas is insufflated. The risk of mediastinal emphysema from gas insufflation has made surgeons wary of endoscopic neck surgery. To create sufficient working space, we have used a neck region lifting method [3–5] that eliminates the need for gas insufflation.

In the past 5 years, surgeons at over 30 institutions in Japan including our department have performed endoscopic neck surgery. Some of the associated difficulties have been addressed by recent technical improvements. However, many problems remain to be solved. Of particular importance is a lack of concerns advantages of endoscopic neck surgery for patients with

*Corresponding author. Current address: Department of Otolaryngology, Head and Neck Surgery, Faculty of Medicine, Tottori University, 36-1 Nishimachi, Yongao, Japan 683-8504. Tel.: +81-859-34-8123. Fax: +81-859-34-8090. E-mail: hkitano@grape.med.tottori-u.ac.jp thyroid tumors. Some surgeons believe that the minimally invasive nature and superior cosmetic results of endoscopic neck surgery may make it preferable to conventional thyroid tumor surgery. Further experience will be necessary to resolve this issue.

In particular, indications in malignant thyroid tumors have not been definitively established. Usually a thyroid mass less than 1 cm in diameter is not treated with an open procedure, while masses only slightly larger than 1 cm and have no apparent metastasis nevertheless are managed with open surgery, as is the case for large tumors. In such borderline situations, patients' anxiety about malignant disease is compounded by anxiety concerning cosmetic results. For these small thyroid cancers and lesions suspected of malignancy, we now use endoscopic neck surgery.

Here, we describe our present endoscopic neck surgical procedure for malignant thyroid carcinoma, and the results in five patients.

### CASES AND METHOD

### Cases

All five patients with definite or suspected thyroid carcinoma presented here were female; ages ranged from 18 to 63 years old. The 63-year-old patient represented an exception to our age criterion for endoscopic surgery in cancer, but she nevertheless chose endoscopic neck surgery.

Possible complications of endoscopic neck surgery such as recurrent nerve palsy, surgical emphysema and others were discussed with patients under the standard protocol established by the Ethical Committee of Shiga University of Medical Science.

After patients made an informed choice of endoscopic rather than conventional surgery, written consent was given.

### Surgical Procedures

#### *Incisions*

Endoscopic neck surgery was performed under general anesthesia with the patient in the supine position. The neck was extended, and the head was not rotated to either side.

In distinction to endoscopic surgery for benign tumors, the present procedure was performed for small thyroid cancers or lesions suspected to be malignant, involved three skin incisions in the anterior chest and axillary fosse to allow more easy access to the neck (Fig. 1). These incisions shown in Fig. 1 are as a standard incisions for endoscopic neck surgery, and we modified the positions of incision according to the tumor location and/or lymph nodes swelling. These incisions were positioned so scars would be covered by the patients' undergarments. Though axillary incision, a 12-mm trocar was passed, and through this trocar a 10-mm endoscope with a video camera was inserted. Through the other two incisions, one in axillary fosse ipsilateral to the tumor and one in the anterior chest, 12-mm trocars were passed. As needed, one or two other additional incisions were made in the precordial region for 3-mm trocars. Through these trocars the surgical instruments were inserted. As our method does not involve insufflation of carbon dioxide to create an open space, endoscopic the surgical instruments were advanced through these trocars to avoid injury from instruments catching on subcutaneous tissue.

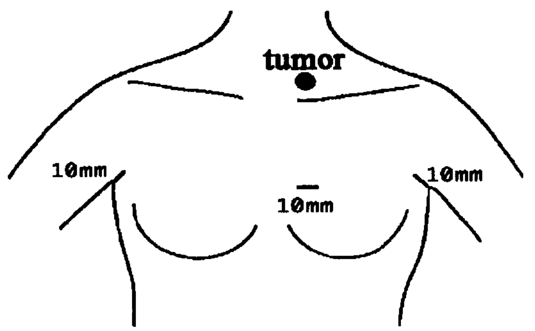

FIGURE 1 A schema of incisions for endoscopic neck surgery. The shadow area represents the area dissected under the skin.

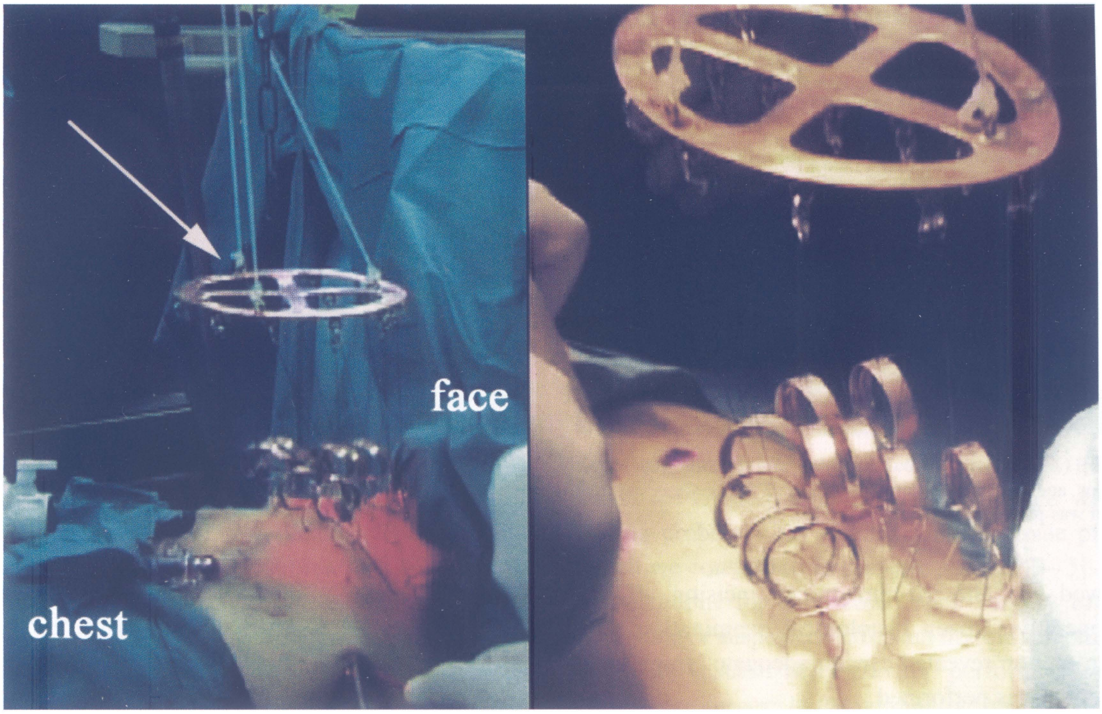

FIGURE 2 The neck skin over the thyroid mass was lifted by hooks (Case No. 3). The white arrow indicates our device for lifting the neck skin.

### *Creating Working Space*

Blunt dissections were carried out under the skin of the chest toward the thyroid gland. Skin overlying the tumor was lifted by a special designed device (Japanese Patent No. 3049394), neck region lifting method (Fig. 2) [3–5]. An open space was created just beneath of the platysma muscle. Next, the sternocleidomastoid muscle, strap muscles, and internal jugular vein were identified. The sternocleidomastoid muscle was drawn laterally using vessel tape. The sternothyroid muscle and sternohyoid muscle were medially as shown in Fig. 3.

### *Resection of Masses*

Using these procedures above, the thyroid was easily identified. Superior and inferior thyroid arteries and veins were cut using a 10-mm Harmonic scalpel (HS) (Johnson–Johnson Medical, Cincinnati, OH). The thyroid isthmus also was cut using the HS (Fig. 4). We do not use haemostatic-clips when cutting the thyroid arteries. In all cases the recurrent laryngeal nerve was identified and preserved (Fig. 5), was facilitated by use of special bipolar scissors (Fig. 5). The tumor was cut into pieces, which were in a plastic bag and

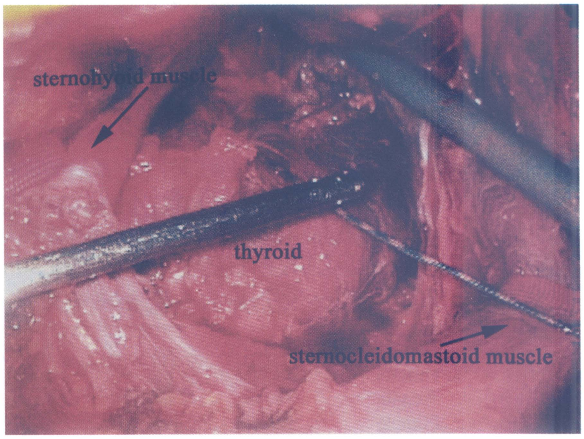

FIGURE 3 Operative view provided by a 10-mm endoscope. Sternocleidomastoid muscle was drawn laterally and sternohyoid muscle was drawn medially by vessel tape (Case No. 3).

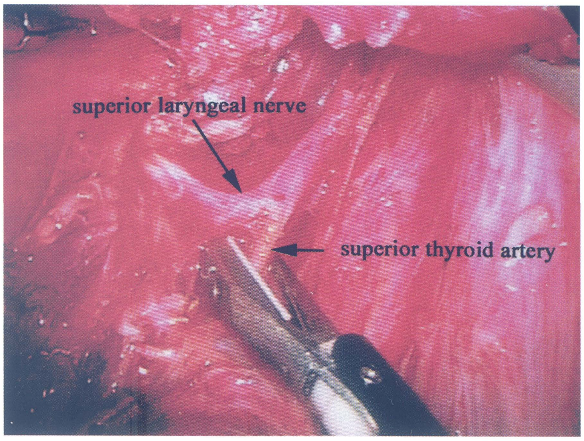

FIGURE 4 Operative view provided by 10-mm endoscope. Superior and inferior thyroid arteries and veins were cut and thyroid was pulled up medially (Case No. 3).

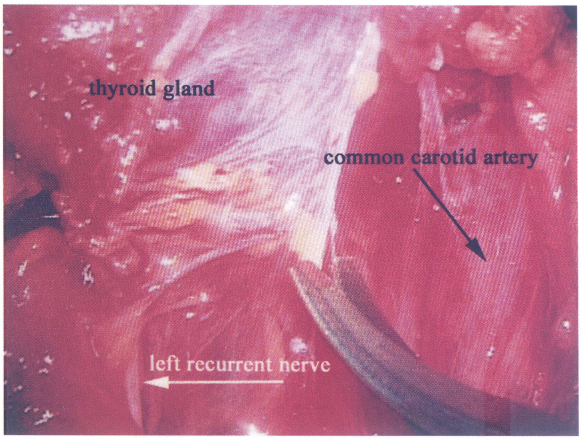

FIGURE 5 Operative view provided by 10-mm endoscope. The arrow indicates the recurrent laryngeal nerve. Special bipolar scissors were used to preserve nerve (Case No. 3).

removed via the 10-mm trocar incision. If the intraoperative frozen histological examination of a frozen section revealed cancer, adequate lymph node dissection was performed.

### *Postoperative Care and Course*

A suction drain (5 mm) initially left in place was removed on the following day. In some cases a self-limited low-grade fever occurred for a few days in patients with wide dissection of the precordial region. Postoperative scars in the precordial region were covered by undergarments, and all patients were satisfied with the cosmetic result (Fig. 6).

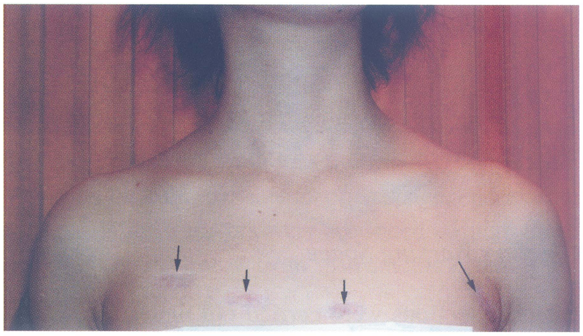

FIGURE 6 Post-operative result (2 months after surgery). The incisions scars were small and completely covered by the patients' undergarments (Case No. 3).

### RESULTS

Patient profiles are shown in Table I. Histological examination was diagnostic for thyroid cancer in five

TABLE I Case profile in endoscopic neck surgery for thyroid cancer (p/c, papillary carcinoma; ade, adenoma; f/c, follicular carcinoma; sub, subtotal thyroid lobectomy; lob, thyroid lobectomy; isth, thyroid isthmusectomy; small, blood logs under 30 ml)

| Case No. | Gender | Age (years) | Disease | Tumor size (cm) | Procedure | Time of operation (min) | Blood loss volume (ml) |
|---|---|---|---|---|---|---|---|
| 1 | Female | 63 | p/c | 2.3×2.2 | sub | 431 | small |
| 2 | Female | 34 | p/c | 2.0×1.5 | sub | 300 | 185 |
| 3 | Female | 18 | p/c | 2.0×1.5 | lob+isth | 503 | small |
| 4 | Female | 45 | ade | 2.0×1.8 | lob+isth | 280 | small |
| | | | p/c | 0.5×0.5 | – | – | – |
| 5 | Female | 38 | f/c | 1.3×1.4 | lob+isth | 230 | small |
| | | | ade | 0.5×0.3 | – | – | – |

patients, with associated benign lesions in two. Thyroid lobectomy and isthmusectomy were performed in three cases, while subtotal lobectomy was performed in two. In three cases of papillary carcinoma, central lymph nodes were dissected including paratracheal, tracheoesophageal, and criocothyroid as well as superior mediastinal lymph nodes in the immediate area of surgery. In case 3, frozen section histological examination revealed lymph node metastasis, and as a result modified neck dissection was added. In case 4, a 5-mm carcinoma was found incidentally during surgery for a larger tumor that proved to be an adenoma, and no lymphadenopathy was found during the operation. In case 5, intraoperative frozen-section histological examination indicated a follicular adenoma, but subsequent histological examination of paraffin sections was diagnostic of follicular carcinoma; therefore lymph node dissection could not be performed.

Intraoperative blood loss for each case is shown in Table I. Loss was relatively great in case 2 in which hemorrhage occurred from the external jugular vein. Duration of the entire surgical procedure ranged from 230 to 530 min (mean, 381.). No major surgical complications were noted. Temporary recurrent nerve palsy occurred in one case. In all cases where surgical emphysema occurred, it was subcutaneous and limited to the neck, disappearing in a few days. As mentioned previously, incisions were completely covered by undergarments and patients were satisfied with cosmetic results. And the course of recovery from surgery was considered excellent in all cases.

### DISCUSSION

Endoscopic neck surgery has several advantages over conventional neck surgery, including results [6–11]. Future innovations in instruments eventually will allow endoscopic neck surgery to be performed by general surgeons. The technique that we presently employ requires incisions for introduction of the video, endoscope and surgical instruments. These incisions are placed so that scars will be hidden by the patients' undergarments, leaving no scar in the neck. In addition, since skin and muscles in the neck are not cut in endoscopic neck surgery, patients do not experience the uncomfortable sensations associated with skin or muscle contraction after surgery. The small size of incisions also facilitates early post-operative recovery.

The greatest difficulties in about endoscopic surgery involve the small size of the working space, which is created by low-pressure carbon dioxide insufflation [6] or lifting the skin of the neck. The main risk carried by insufflation is pneumomediastinum. Instead of insufflation, we lifted the skin of the neck using special designed device (Fig. 2) [3–5]. Thus, we created sufficient working space without risking problems gas leakage through tissue planes, which we considered in previous reports [3–5].

Indications for endoscopic neck surgery to treat malignant or suspected malignant thyroid tumors have not been established. Having performed video-endoscopic surgery in the present five cases of thyroid carcinoma, we presently advocate these criteria:

1. Women under 45 years old.
2. Tumor size from 0.5 to 2 cm.
3. No apparent lymph node metastasis by ultrasonography and computed tomography (CT).
4. No tumor invasion of surrounding tissues according to ultrasonography and CT.
5. Informed patient choice.

The most important point to be made about endoscopic neck surgery for malignant lesions is that we can perform resection radical lymph nodes dissection as we perform resection and lymph node dissection to the same extent as in conventional surgery (i.e. equivalent radicality). Nonetheless, further experience will be needed to establish this method as a standard procedure for young female patients with malignant thyroid lesions.

### *References*

[1] Gagner, M. (1996) "Endoscopic subtotal parathyroidectomy in patients with primary hyperparathyroidism", *Br. J. Surg.* **83**, 875.

[2] Naitoh, M., Gagner, M., Garcia-Ruiz, B., *et al.* (1997) "Endoscopic endocrine surgery in the neck", *Surg. Endosc.* **12**, 202–205.

[3] Kitano, H., Fujimura, M., Hirano, M., *et al.* (2000) "Endoscopic surgery for a parathyroid function adenoma resection with the neck region-lifting method", *Otolaryngol. Head Neck Surg.* **123**, 465–466.

[4] Kitano, H., Fujimura, M., Hirano, M., *et al.* (2000) "Endoscopic surgery for lateral cervical cysts", *Surg. Endosc.* **14**, 1086.

[5] Kataoka, H., Kitano, H., Fujimura, M., *et al.* "Endoscopic resection of Zenker's diverticulum. A case report", *Diagn. Therap. Endosc.*.

[6] Brunt, L.M., Jones, D.B., Wu, J.S., *et al.* (1997) "Experimental development of an endoscopic approach to neck exploration and parathyroidectomy", *Surg.* **122**, 893–901.

[7] Yeung, G.H.C. (1998) "Endoscopic surgery of the neck", *Surg. Laparosc. Endosc.* **8**, 227–232.

[8] Ishii, S., Ohgami, M., Arisawa, Y., *et al.* (1998) "Endoscopic thyroidectomy with precordial approach", *JSES* **3**, 159–163.

[9] Takami, H., Ikeda, Y. and Wada, N. (2000) "Surgical management of primary hyperparathyridixsm", *Biomed. Pharmacother.* **54**, 21–24.

[10] Chowbey, P.K., Mann, V., Khullar, R., *et al.* (1999) "Endoscopic neck surgery: expanding horizons", *J. Laparoendosc. Adv. Surg. Tech.* **9**, 397–400.

[11] Shimizu, K., Shigeno, A. and Tanaka, S. (1998) "Video assisted neck surgery: endoscopic resection of benign thyroid tumor aiming at scarless surgery on the neck", *J. Surg. Oncol.* **69**, 178–180.

